# Improving the Angular Visibility of Photopolymer-Based Reflection Holograms for Sensing Applications

**DOI:** 10.3390/s23094275

**Published:** 2023-04-25

**Authors:** Tatsiana Mikulchyk, Kevin Murphy, John Walsh, Suzanne Martin, Dervil Cody, Izabela Naydenova

**Affiliations:** 1 Centre for Industrial and Engineering Optics, School of Physics, Clinical and Optometric Sciences, Faculty of Sciences and Health, Technological University Dublin, Grangegorman, D07 H6K8 Dublin, Ireland; 2 School of Art and Design, Faculty of Arts and Humanities, Technological University Dublin, Grangegorman, D07 H6K8 Dublin, Ireland

**Keywords:** holographic sensor, pressure-sensitive photopolymer, pressure sensor, laser speckle pattern, reflection grating, speckle grating

## Abstract

Volume reflection hologram-based sensors are designed to visibly change colour in response to a target stressor or analyte. However, reflection holograms fabricated in thick photopolymer films are highly angularly selective, making these sensors challenging to view and interpret by non-experts. Here, the use of speckle holography to improve the visibility of reflection holograms is presented. A novel recording approach combining speckle recording techniques with Denisyuk reflection recording geometry is described. The recorded speckle reflection grating operates as a series of multiplexed reflection gratings with a range of spatial frequencies, capable of reflecting light at a wider range of angles. A comparative study of the angular and wavelength selectivity of speckle and standard reflection gratings was conducted. The FWHM of the angular selectivity curves of the speckle reflection gratings is doubled (4°) in comparison to standard 4500 lines/mm reflection gratings (2°). The wavelength selectivity FWHM is also doubled from 4.2 to 8.6 nm. The comparative ability of the speckle and standard reflection gratings to act as colour-changing compressional pressure sensors in the 0.88–5.31 MPa range is described. Finally, we present a prototype reflection hologram viewer which enables the easy observation of angularly specific reflection holograms by non-experts.

## 1. Introduction

Volume reflection holograms fabricated in photopolymer media have been widely investigated for the holographic sensing of both physical stressors and chemical analytes, including environmental temperature and relative humidity [1,2], pH [3], glucose [4], and others [5,6,7]. The interest in reflection holograms as a sensor transduction mechanism is due largely to the fact that the reconstructed reflection hologram, once correctly functionalised, visibly changes colour in response to the intended physical stressor or chemical/biological analyte. The change in the reconstructed hologram colour (i.e., wavelength) arises due to a change in the grating’s average refractive index and/or periodicity, as a direct result of exposure to the stressor or analyte. Such a sensor which relies on a visible colour change is operationally simple, effective, and easily interpretable by non-expert users, requiring little to no training. The potential societal impact of reflection hologram-based sensors is significant due to their wide applicability across a multitude of sectors, including medical diagnostics (in situ blood testing, saliva testing, etc.), the real-time monitoring of environmental contaminants (both airborne and water-based), smart food packaging to minimize food waste, and authentication of high importance documents and luxury goods.

Photopolymer films are an attractive option for holographic sensors which rely on dimensional and average refractive index-based changes in the volume grating, as they are flexible and elastic, and can be selectively functionalised for analytes using nanoparticles and other chemical agents [8]. Several studies have successfully demonstrated the use of elastic photopolymer-based reflection gratings as transducers for pressure-sensing applications. The application of mechanical pressure to the reflection grating results in a reduction in the grating fringe spacing as well as a simultaneous increase in the film’s average refractive index, thereby shifting the wavelength of reconstructed light from the grating in accordance with Bragg theory. A study by Cody et al., reported a sensitivity to a compressional pressure of 7 nm/GPa for reflection gratings recorded in a low toxicity diacetone acrylamide-based photopolymer [9]. Liu et al., have investigated the compressional pressure-sensing potential of slanted reflection gratings in PQ-PMMA photopolymers and report a sensitivity of 5.2 × 10^4^ Pa/nm [10]. The use of reflection gratings fabricated in a haloalkane–acrylate photopolymer mixture for compression and rotation sensing was recently reported by Castagna et al., a rotational sensitivity of 159 nm/rad is reported [11]. Similar studies have reported on the suitability of acrylate-based transmission and reflection gratings for the quantification of both tensile and bending pressures [12,13].

**Figure 1:**
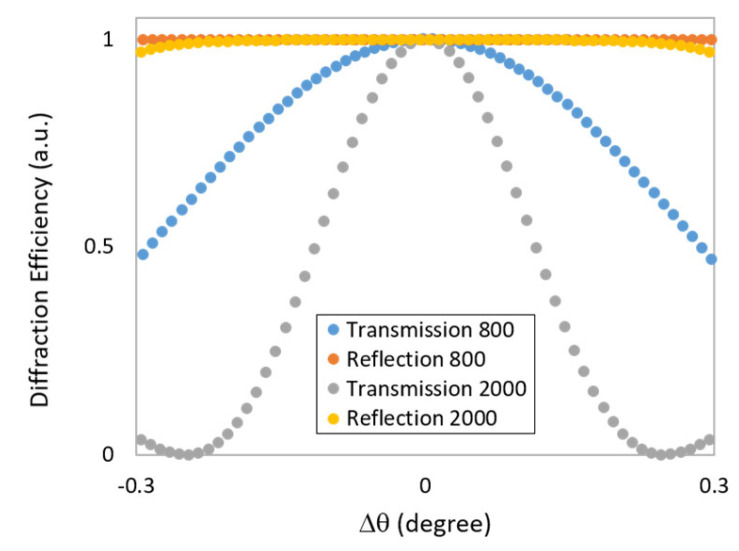
Angular selectivity of transmission and reflection gratings with spatial frequencies of 800 and 2000 lines/mm and 100 μm thickness. Simulation was performed by the use of Kogelnik theory described in Section 2.1.

The rule of thumb in holography is that reflection holograms are highly wavelength-selective but less angularly selective; the opposite is true for transmission holograms [14,15]. The comparative angular selectivity of both transmission and reflection gratings predicted using Kogelnik’s coupled wave theory [15] is illustrated in Figure 1 for gratings with spatial frequencies of 800 and 2000 lines/mm (100 μm grating thickness). The low angular selectivity of the reflection holograms means that they should be readily visible by an observer. However, the angular selectivity will depend on the specific grating parameters (spatial frequency, refractive index modulation, and thickness) which in turn will depend on the intended application of the grating. Photopolymer films for display reflection holography are generally on the thinner side (20 µm); this improves the angular visibility (i.e., less angularly selective) and in addition helps to minimise the differences in the two recording beam intensities during the recording process, where the two beams are incident from the opposite sides of the sample. Certain applications, however, require thicker photopolymer films and gratings. In the case of reflection grating-based pressure sensors, the photopolymer films must be robust and/or thick if they are to withstand high mechanical pressures, and >100 µm thicknesses are regularly reported [9,12,13]. Moreover, independent studies by both Liu et al. [10] and Cody et al. [9] have concluded that photopolymer film thickness can be varied in order to tune the pressure sensitivity of the sensor, due to the relationship between film thickness and bulk elasticity. Other applications which require thick (>100 µm) photopolymer film-based reflection gratings include narrow-bandwidth holographic reflection filters [16]. While necessary, the increased film thickness has the undesired effect of increasing the angular selectivity of the recorded reflection gratings, making them harder to see and, in the case of visual colour-changing sensors, harder to interpret, particularly by non-experts.

The incorporation of diffusers by recording holograms of microstructures [17,18] or recordings by other optical devices such as retroreflectors in holograms [19] have been explored as a means of increasing the angle of view. However, these additional devices inevitably decrease the intensity of the diffracted light in any given direction. To counteract this effect, it is necessary to use holograms with high diffraction efficiency.

Here, we present a novel approach for improving the visibility of angularly selective reflection holograms using a reflection mode speckle recording technique. Speckle patterns are extensively exploited in speckle imaging and metrology techniques [20,21,22,23] as well as in the fabrication of optical diffusing elements that provide multiple scattering and beam shaping [24,25]. To fabricate the speckle holograms, the coherent beam is passed through optically rough surfaces, or in a random medium, and creates a speckle pattern, which produces the ‘speckle’ features of a modulated refractive index and of varying size inside the recording medium [26,27]. Recently, a new method for fabricating holographic beam-shaping diffractive diffusers by holographic recording a laser speckle pattern with controlled speckle size and shape has been reported [25]. This method, successfully demonstrated in an acrylamide-based holographic photopolymer, has allowed for the development of diffractive diffusers operating in transmission mode with a regulated output beam shape and angular size.

Here, the fabrication as well as the angular and wavelength characterization of speckle reflection gratings in a diacetone acrylamide-based photopolymer is presented. The angular visibility of the speckle reflection gratings, quantified as the full width at half the maximum intensity (FWHM) of the angular selectivity curves, is observed to double in comparison to the standard reflection gratings, for the same material and film thickness. Furthermore, the ability of speckle reflection gratings to act as visual colour-changing mechanical pressure sensors is demonstrated for mechanical compressional pressure in the 0.881–5.31 MPa range. Finally, the design and fabrication of a prototype reflection hologram viewer is reported. The viewer enables the easy observation of angularly specific reflection holograms by non-experts and further opens the door for the development of refractive index-based optical sensors for a multitude of applications.

## 2. Theory

### 2.1. Phase Reflection Gratings

Phase reflection holograms (henceforth referred to as standard reflection gratings) are recorded in photosensitive materials when the spatially varying intensity created by the two (or more) interfering beams is copied in the form of refractive index variation. In this case, the two recording beams approach the material from the two opposite sides of the photosensitive film; typically, the period of the created structure is sub-micron.

In accordance with Kogelnik’s theory [15], the diffraction efficiency η of a reflection hologram of thickness *d* is given by:

(1) $$\eta =\frac{1}{\left\{1+\frac{\left(1-\frac{\xi^{2}}{\nu^{2}}\right)}{\sinh^{2}{\left(\nu^{2}-\xi^{2}\right)}^{\frac{1}{2}}}\right\}}$$

where the parameters ξ and v are, respectively, given by:

(2) $$\xi =\frac{\Delta \theta Κd\sin\left(\theta_{0}-\phi \right)}{2c_{s}}=\frac{\Delta \lambda {Κ}^{2}d}{8\pi nc_{s}}$$

(3) $$v=\frac{\pi n_{1}d}{\lambda {\left(c_{R}c_{x}\right)}^{\frac{1}{2}}}$$

In these expressions, Δθ is the angular deviation, Δλ is the wavelength deviation, K is the grating vector, $\theta_{0}\;$is the grating Bragg angle, ϕ is the grating slant angle, $n_{1}$ is the refractive index modulation, and λ is wavelength.

The obliquity factors $c_{R}$ and $c_{S}\;$are given by $c_{R}=\cos\theta$ and $c_{s}=\cos\theta -\frac{K}{\beta }\cos\phi \;$, where β is the free propagation constant.

For unslanted reflection gratings (i.e., $\phi =0^{0}$) at Bragg incidence, $c_{s}=-c_{R}=-\cos\theta_{0}$ and Equation (1) becomes:

(4) $$\eta =\tanh^{2}\left(\frac{\pi n_{1}d}{\lambda \cos\theta_{0}}\right)$$

**Figure 2:**
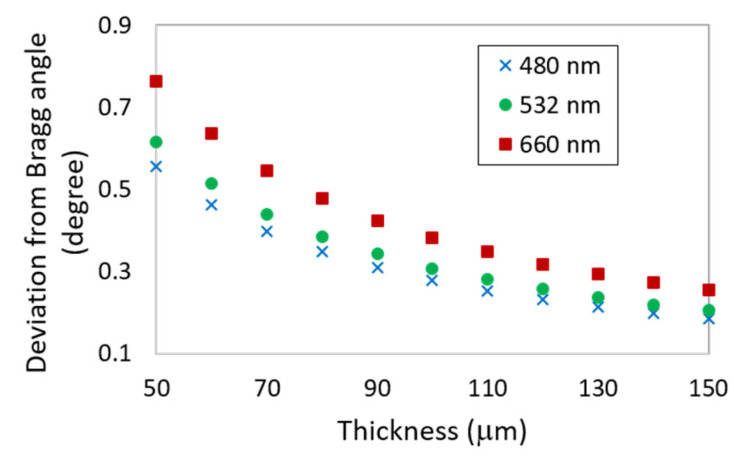
Deviation from the Bragg angle (Δ*θ*) required for the diffraction efficiency of a reflection grating (initial *η* = 35%) to fall to 0% vs. the reflection grating thickness for reconstruction wavelengths of 480, 532, and 660 nm.

Figure 2 shows the theoretically predicted angular deviations from the Bragg angle, at which the diffraction efficiency of a standard reflection grating (initial η = 35%) will drop to zero, for grating thicknesses in the range of 50–150 µm and at three different wavelengths. The required deviations are less than a degree in all cases, clearly showing the angularly selective nature of the reflection gratings.

### 2.2. Speckle Reflection Gratings

A single-beam holographic method for creating transmission holographic diffusers with beam shaping properties has previously been reported [25]. The optical recording set-up for this method consists of a collimated laser beam which propagates through a ground glass diffuser, with an adjustable aperture in the same plane, before being focused onto a sample of photosensitive material placed in the focal plane of a focusing lens. The beam shaping properties of the transmission diffusers are determined by the size and shape of the speckles recorded in the photosensitive recording medium, which in turn are controlled by the wavelength of the recording laser *λ*, the size of the adjustable aperture *a*, and the focal length of the focusing lens *f_fl_*. In the case of subjective speckle, where a speckle pattern is imaged by a lens, the (finite) lens aperture determines the minimum size of speckle that is displayed in the focal plane of the lens. Through the accurate control of the adjustable aperture, a high degree of precision can be achieved in determining the range of speckle sizes in the lens focal plane. In the subjective speckle case for transmission holographic diffusers, the minimum speckle size *σ* is given by:

(5) $$\sigma =\frac{2.44\lambda f_{fl}}{a}$$

According to Equation (5) and estimations from phase contrast microscope images [25], the speckle size for transmission speckle gratings is in the range of 10–50 μm.

Here, this method for creating holographic diffusers is extended into the reflection hologram regime by combining the single beam transmission recording method with a standard Denisyuk geometry reflection configuration. It is theorized that a speckle reflection grating comprised of large speckle grains will have reduced angular selectivity (i.e., larger FWHM values) than standard reflection gratings. Unlike standard reflection gratings which contain one spatial frequency only, speckle reflection gratings contain a broader range of spatial frequencies as well as a range of grating slant angles. This will serve to increase the FWHM of the angular selectivity curves, and therefore improve the visibility of the speckle reflection gratings. The speckle size for a reflection diffuser created in this set-up would be smaller than that in transmission recording, assuming a mirror and a sample of infinite diameter, with the mirror placed directly behind the recording medium. This also assumes that the speckle fields will not be decorrelated from each other. In practice, the sample and mirror size will be finite, and the mirror is at a finite distance from the sample. In this case, the size and shape of the speckles created will be determined by the path length from the sample to the mirror and back to the sample and the diameter of the aperture/pupil of the recording medium.

## 3. Materials and Methods

### 3.1. Preparation of Photopolymer Solution and Films

Photopolymers undergo free radical polymerisation upon exposure to light of specific wavelengths for which they are photosensitized. The photoinitiated polymerisation of monomer molecules into polymer chains induces a localised change in the refractive index and/or density of the photopolymer film in illuminated regions only. This allows for the permanent inscribing of periodic structures such as diffraction gratings in the photopolymer film. A low-toxicity diacetone acrylamide photopolymer was previously optimised for reflection mode holographic recording via the incorporation of a chain transfer agent (citric acid) and a free radical scavenger (glycerol) [28]. The purpose of both additives is to restrict the growth and movement of polymer chains from illuminated to non-illuminated regions, which diminishes the refractive index modulation and thus the diffraction efficiency of the grating. For this study, the diacetone acrylamide-based photopolymer composition was minimally modified via the replacement of citric acid with lactic acid. The purpose of the replacement was to reduce the humidity sensitivity of the photopolymer films, as the chain transfer agent citric acid is highly hygroscopic, and it was observed that the dry film optical quality degraded over time due to water absorption from the environment. Lactic acid was chosen as a replacement chain transfer agent, as it also can act as a chain transfer agent [29] and has lower hygroscopicity than citric acid [30].

The photopolymer composition used consists of a binder (polyvinyl alcohol, 20 mL, 10 *w*/*v*), monomer (diacetone acrylamide, 1 g), cross-linker (*N*,*N*-methylene bisacrylamide, 0.2 g), free radical generator (triethanolamine, 2 mL), plasticiser and free radical scavenger (glycerol, 2 mL), chain transfer agent (lactic acid, 0.075 g), and sensitizing dye (Methylene Blue, 3 mL, 0.11% *w*/*v*). An IKA^®^ C-MAG HS magnetic stirrer was used to ensure the thorough mixing of the photopolymer components in two stages. First, the monomer, cross-linker, free radical generator, and chain transfer agent were added to the binder solution and mixed for 1.5 h. The plasticizer and sensitizing dye were then added and the entire solution was mixed for 1 hour, at which point the solution was ready to use. Photopolymer layers with a thickness of 150 ± 5 μm were prepared by the deposition of 0.75 mL of solution onto the levelled glass slides (26 × 76 mm^2^) and dried for 24 h in a dark room at T = 21 ± 2 °C and RH = 30 ± 5%.

### 3.2. Holographic Recording of Standard Reflection Gratings

**Figure 3:**
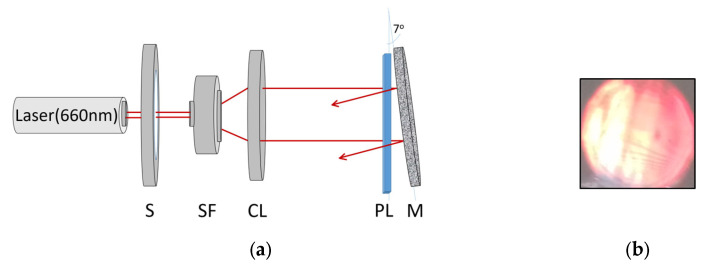
(**a**) Experimental set-up for the holographic recording of standard reflection gratings with Denisyuk recording geometry: S—electronic shutter; SF—spatial filter; CL—collimator; PL—photopolymer layer; M—mirror; and (**b**) Photograph of the volume phase reflection grating under illumination with a broadband source. The grating selectively diffracts light with the wavelength in the red region of the spectrum.

The reflection volume phase gratings were recorded using a Denisyuk reflection geometry, as shown in Figure 3a. All recordings were carried out on an optical table (Newport RS 4000), which was floated in order to provide mechanical stability for the optical set-up. Reflection gratings with a spatial frequency of 4500 lines/mm were recorded at a 660 nm wavelength using a Cobolt Flamenco 500 laser. The intensity of the recording beam was 10 mW/cm^2^ and the recording time was 100 s. After holographic recording, the created photonic structures were UV-cured using a Dymax UV curing system with a total exposure of 5.4 J/cm^2^. Samples were then laminated with a Melinex® cover sheet (50 µm-thick). Figure 3b is a photograph of a typical volume phase reflection grating recorded in the diacetone acrylamide photopolymer under illumination with a broad band light source. The on-Bragg diffraction efficiency of the fabricated standard reflection gratings was measured using a 660 nm probe laser and calculated as the ratio of the intensity of light diffracted in the first order to the intensity of the incident probe beam. Accounting for Fresnel reflection losses in the incident 660 nm beam, the diffraction efficiency was found to be 35 ± 2%.

### 3.3. Holographic Recording of Speckle Reflection Gratings

**Figure 4:**
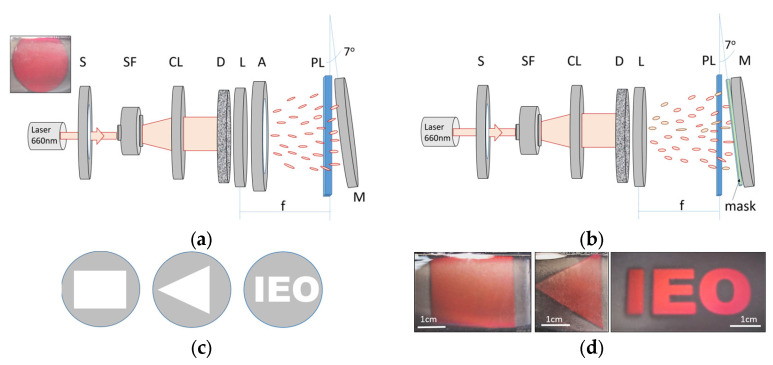
(**a**) Experimental set-up for the holographic recording of speckle reflection gratings with no mask on the mirror producing an object recording beam. The insert shows the photograph of the image produced by the speckle grating under illumination with a broad band light source; (**b**) Experimental set-up for the holographic recording of speckle reflection gratings using masks attached to the mirror producing an object beam. S—electronic shutter; SF—spatial filter; CL—collimator; D—diffuser; L—focusing lens; A—aperture; PL—photopolymer layer; M—mirror; f—focal length; (**c**) templates of masks; (**d**) Photographs of the speckle gratings recorded using masks with the shape of a square, triangle, and the text ‘IEO’. Photographs were taken under illumination with a broadband light source.

In the case of the speckle reflection gratings, the photopolymer layers were laminated with a Melinex^®^ cover sheet (50 µm thick) prior to recording in order to avoid the formation of surface structures previously observed during the holographic speckle patterning of photopolymers [31]. A schematic presentation of the holographic speckle recording set-up with Denisyuk recording geometry is shown in Figure 4a. As discussed in Section 3.2, a Cobolt Flamenco 500 (λ = 660 nm) laser beam passes through a spatial filter and is collimated by a lens. The beam then propagates through a ground glass diffuser (220 grit polish) generating a speckle pattern. The photopolymer layer is placed at the focal plane of the focusing lens (f = 10 cm). Passing the photopolymer layer, the diffused beam is reflected onto the photopolymer layer. As a result, a speckle reflection grating is fabricated in the volume of the photopolymer layer. The recorded structure is assumed to be a randomly distributed set of Bragg reflection gratings, each having the dimensions of a single speckle. The on-Bragg diffusion efficiency of the fabricated speckle reflection gratings was measured using a 633 nm probe laser and calculated as the ratio of the intensity of light diffused in reflection to the incident 633 nm beam intensity. Correction for Fresnel losses for the incident beam was considered and the intensity of light transmitted through the device (both zero order and diffused light after the sample) were measured, yielding a diffusion efficiency value of 35 ± 7%. An adjustable aperture at the pupil of the focusing lens is used to vary the speckle size and shape, as discussed previously. During the recording, the photopolymer layers were exposed to a 4 mW/cm^2^ beam for 400 s. After holographic recording, the created photonic structures were UV-cured using a Dymax UV curing system with a total exposure of 5.4 J/cm^2^. To demonstrate the flexibility of the recording approach, speckle gratings were also recorded using masks with the shape of a square, triangle, and the text ‘IEO’ (Figure 4b–d).

### 3.4. Phase Contrast Microscope Imaging

An Olympus BX51 phase contrast microscope, with a DP72 camera (12.8 Mega pixels, 4140 × 3096, with 12-bit resolution) and a 40× magnification was used to visualise the speckle features inside the volume of the reflection grating.

### 3.5. Grating Angular Selectivity Measurements

**Figure 5:**
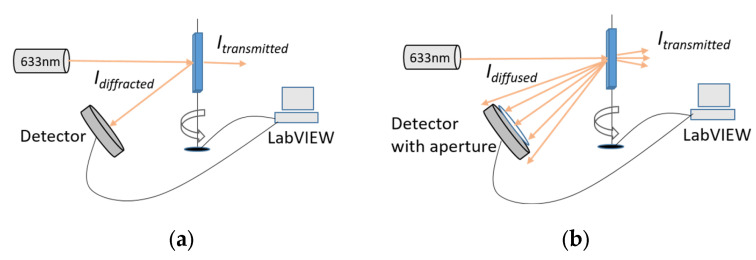
Schematic diagram of the angular selectivity measurement set-up for the standard reflection grating (**a**) and speckle reflection grating (**b**).

The set-ups shown in Figure 5a,b were used to measure the angular selectivity of both the standard and speckle reflection gratings, respectively. The sample was mounted on a rotational stage which was computer-controlled via a motion controller (model Newport ESP300 with angular resolution of 0.001°). A 633 nm laser beam was used as a probe beam. The angular selectivity curve measurement was performed by monitoring the diffracted/diffused beam intensity using an optical power meter (Newport model 840) while the sample was rotated. LabVIEW software was used to acquire and plot the diffraction grating/diffuser efficiency versus the rotational angle in real time. As shown previously, the diffraction efficiency of the reflection grating was calculated as the ratio of the first order diffracted beam intensity (*I_diffracted_*) and transmitted beam intensity (*I_transmitted_*), as shown in Figure 5a. The diffuser efficiency was defined as the ratio of the first-order diffused intensity (*I_diffused_*) and the control zero order intensity (*I_transmitted_*), as shown in Figure 5b. In both cases, the control zero-order intensity or transmitted beam intensity was measured by illuminating a UV-bleached photopolymer layer with no grating.

### 3.6. Grating Wavelength Selectivity Measurements

The wavelength selectivity of both the standard reflection gratings and speckle reflection gratings was measured by use of an Avantes AvaSpec-2048 spectrometer. The probe light from a broadband light source (AvaLight HAL-S) was guided by a fibre optic cable (Avantes FC-UV400-2) to illuminate the diffraction structure. The diffracted light from the reflection grating/speckle reflection grating was coupled through a second fibre optic cable to the spectrometer.

### 3.7. Mechanical Pressure Testing

**Figure 6:**
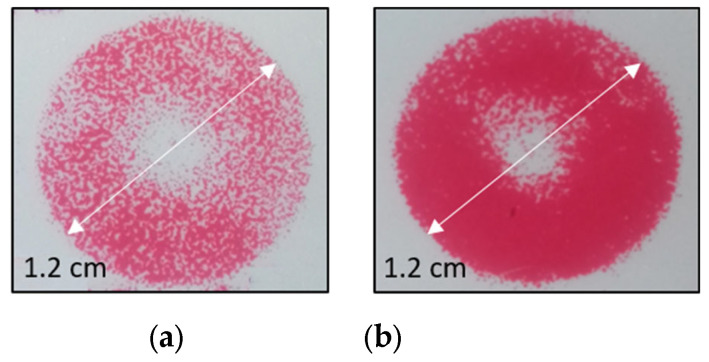
A visual map of the pressure distribution produced for forces of 200 N (**a**) and 500 N (**b**).

The response of both the standard reflection gratings and speckle reflection gratings to applied mechanical pressure was investigated. A tensile tester apparatus (Instron 324 Series 5569 Tensile Tester) was used for these measurements. For each test, a laminated reflection grating sample was first transferred from the glass microscope slide to a plastic substrate (Bayer Makrofol DE1-1cc, 385 µm thick). The sample was then inserted into the tensile tester apparatus and sandwiched between two circular metal presses with a diameter of 1.2 cm (total press area: 1.13 × 10^-4^ m^2^). Forces ranging from 100 to 600 N were applied to each sample. The applied pressure was calculated as the ratio of the force and the sample area. The Fujifilm Prescale Ultra Low (3LW) pressure indicating film was used to provide an independent measurement of the pressure distribution produced by the tensile tester machine. A visual map of the pressure distribution produced for forces of 200 and 500 N is shown in Figure 6. A donut-shaped pressure distribution with an approximately uniform colour density is observed. The spectrum of the light diffracted by each grating was measured before and after the exposure to pressure using the method described in Section 3.5.

### 3.8. Design and Fabrication of Reflection Hologram Viewer

**Figure 7:**
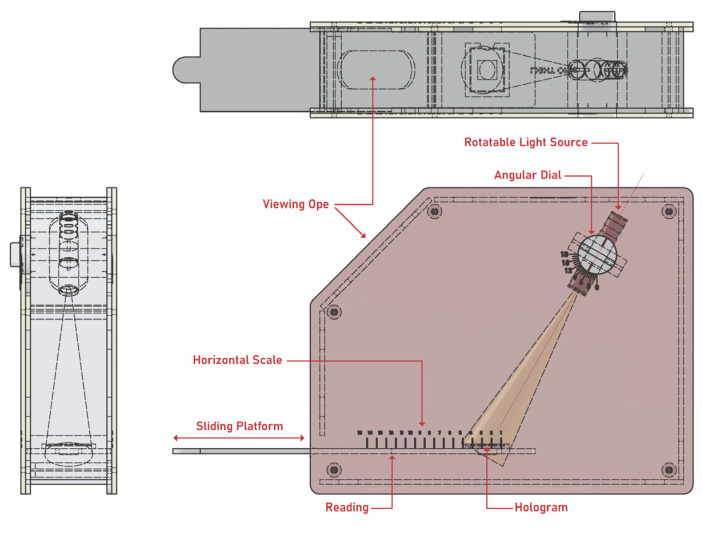
Two-dimensional illustration of different views of the prototype hologram viewer.

**Figure 8:**
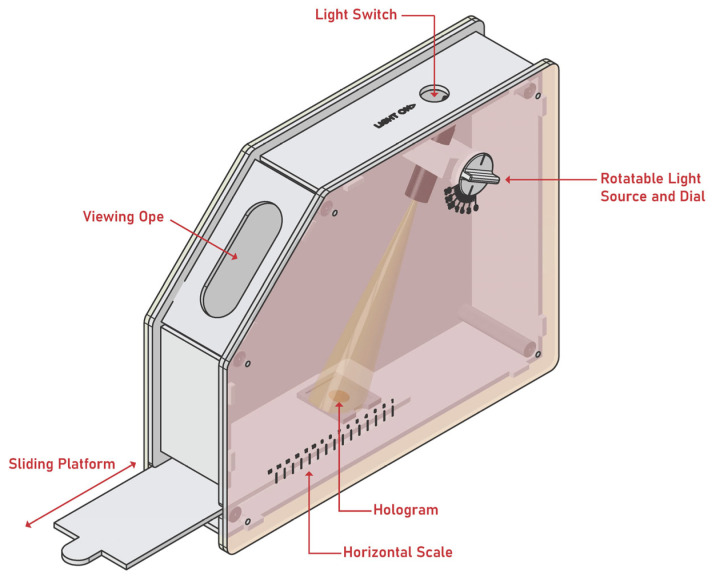
Three-dimensional illustration of the prototype hologram viewer.

The prototype reflection hologram viewer unit was designed using the Autodesk Inventor software and fabricated from a combination of laser cut acrylic sheet and 3D printed Acrylonitrile Butadiene Styrene (ABS) components. The unit, illustrated in 2D and 3D visualisations shown in Figure 7 and Figure 8, respectively, was designed through an iterative process using a combination of rapid prototyping equipment and processes including laser cutting and 3D printing. The operation of the viewer is described in Section 4.4.

## 4. Results

### 4.1. Characterisation of Angular Selectivity of Standard and Speckle Reflection Gratings

**Figure 9:**
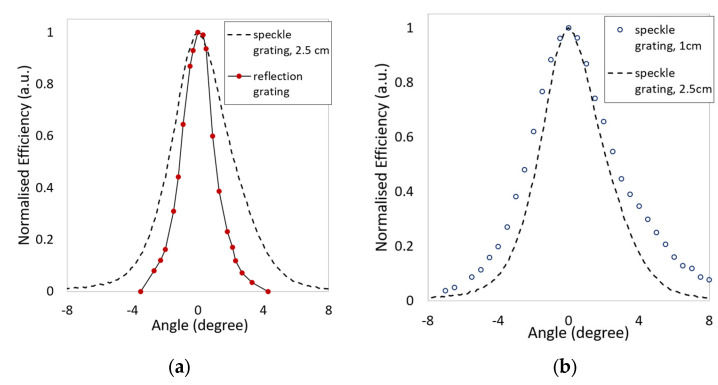
Angular selectivity of the reflection gratings: (**a**) comparison of a standard reflection grating and speckle grating recorded with 2.5 cm aperture; and (**b**) speckle reflection gratings recorded with 2.5 cm and 1 cm aperture (no mask).

Angular selectivity curves for the standard and speckle reflection gratings (two aperture sizes, no mask) are presented in Figure 9. The FWHM values of the curves for the standard and speckle reflection gratings in Figure 9a are calculated to be 2.0° and 4.0°, respectively, demonstrating that the speckle grating is less angularly selective than the standard reflection grating. As previously discussed, the speckle grating represents an assembly of micro-gratings, with a range of spatial frequencies around the average spatial frequency which corresponds to the mean speckle size. Thus, a speckle grating essentially operates as a series of multiplexed reflection gratings with a range of spatial frequencies. Therefore, the increase in FWHM of the speckle gratings is in accordance with Equation (1), which predicts that the angular selectivity depends on the spatial frequency. The fact that the micro-gratings have a range of spatial frequencies is confirmed by the broader wavelength selectivity of the speckle grating in comparison with a standard reflection grating having a single spatial frequency of approximately 4500 lines/mm, as further discussed in Section 4.2.

The influence of the aperture size on the FWHM of the speckle reflection gratings is shown in Figure 9b. The FWHM is slightly increased as the width of the aperture decreases from 2.5 cm to 1 cm. This is as expected: a reduction in aperture size should increase the size of the speckles produced (Equation (5)), which should yield an increased FWHM, i.e., reduced angular selectivity.

**Figure 10:**
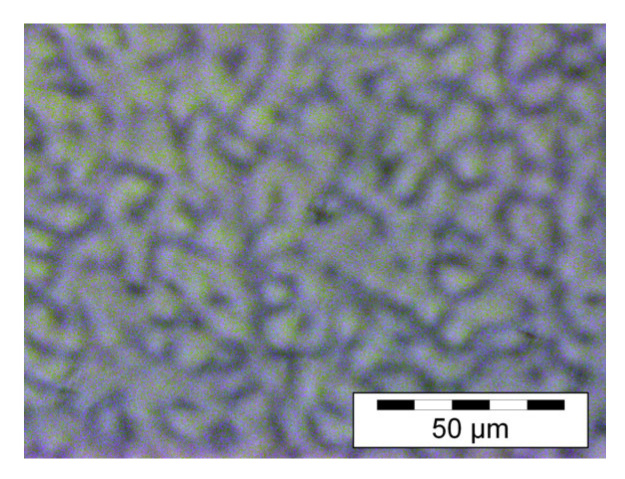
Example phase contrast microscope image of the internal structure of a speckle reflection grating recorded using the method described in Section 3.3.

A sample phase contrast microscopy image illustrating the speckle structure of the reflection speckle grating is shown below in Figure 10. While it is challenging to accurately estimate the size of the speckle, the speckles are similar in dimension to those produced in the transmission regime (10–50 μm [25]).

### 4.2. Characterisation of Wavelength Selectivity of Standard and Speckle Reflection Gratings

**Figure 11:**
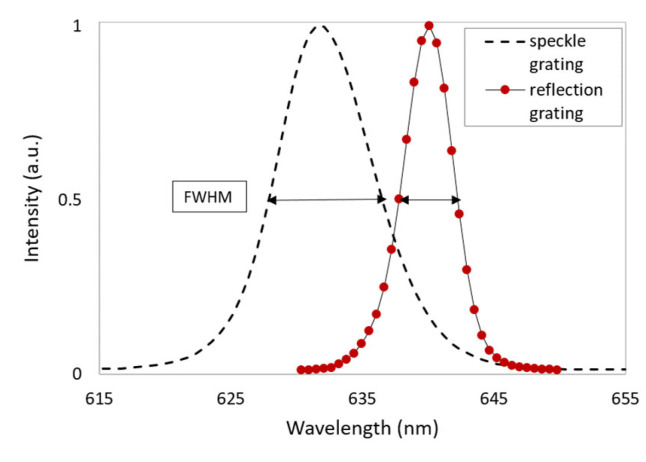
Wavelength selectivity curves of the standard reflection grating and speckle reflection grating recorded with a 2.5 cm aperture.

Wavelength selectivity curves of the standard and speckle reflection gratings were obtained as described in Section 3.6, and the results are presented in Figure 11. The FWHM of the wavelength selectivity curves for the standard and speckle reflection gratings recorded with a 2.5 cm aperture were measured to be 4.2 nm and 8.6 nm, respectively. Like the angular selectivity results, the broader wavelength selectivity curve of the speckle grating may be explained by the wider range of spatial frequencies in the speckle grating in comparison to the single spatial frequency of the standard reflection grating. The broader wavelength selectivity of the speckle grating is beneficial in terms of the visual brightness of holographic devices, as a broader range of wavelengths will be reflected by the grating device when illuminated with a white light source.

### 4.3. Comparative Study of Standard and Speckle Reflection Grating Response to Mechanical Pressure

**Figure 12:**
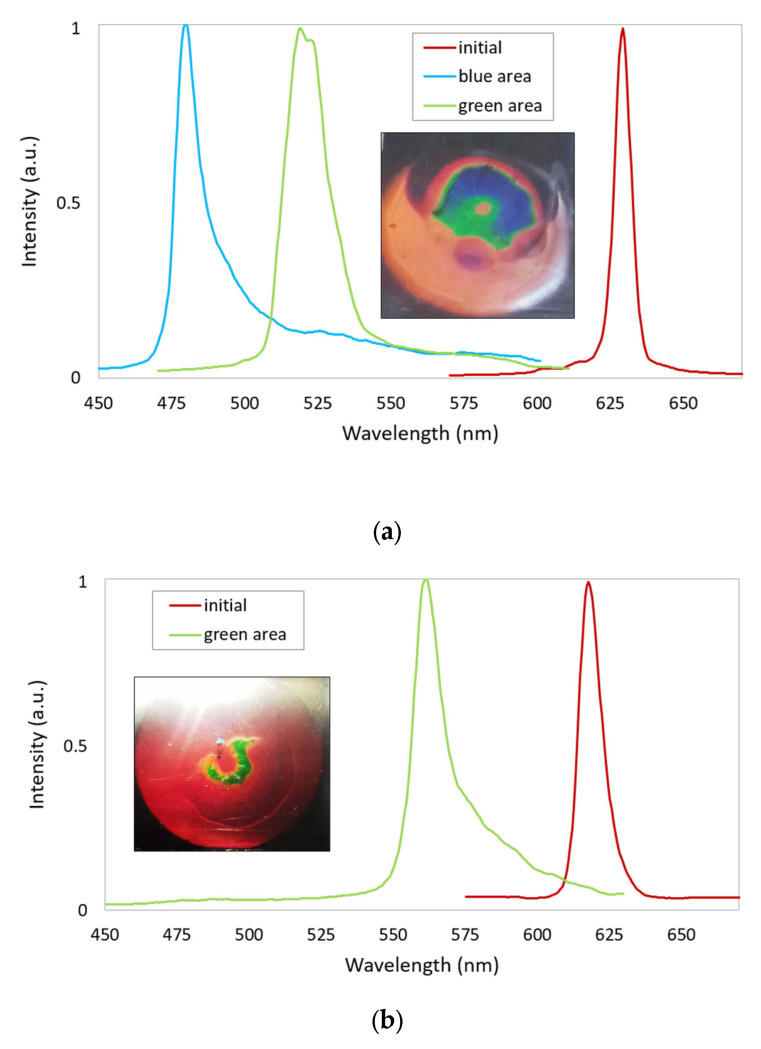
Wavelength selectivity curves of the standard reflection grating (**a**) and speckle reflection grating (**b**) before and after the application to 3.54 MPa pressure. Inserts show the photograph of the gratings after the application to 3.54 MPa pressure.

Both standard and speckle reflection gratings were exposed to compressional mechanical pressure via the method described in Section 3.7. Forces in the range of 100–600 N were applied to both types of grating: this corresponds to pressures of 0.88–5.31 MPa. The results for the produced wavelength shifts at all pressures are summarized in Table 1. Neither the standard nor the speckle grating responded to the lowest tested pressure of 0.88 MPa and both types were damaged by the highest pressure of 5.31 MPa. However, within these threshold limits, a progressively increasing wavelength shift was obtained with increasing compressional pressure for both types of grating. Figure 12 illustrates the blueshift in the spectrum of light reconstructed by standard and speckle reflection gratings before and after a 3.54 MPa pressure application; this is also clearly observable as a visible change in colour. The speckle gratings repeatedly produced a smaller shift in wavelength (18 nm–133 nm) in comparison to the standard reflection gratings (32 nm–168 nm) for the same applied pressures. One possible reason for the reduced pressure sensitivity of the speckle gratings may be the increased rigidity of the speckles themselves which consist of polymerised monomer. Combined with their relatively large size and different morphology, this may make the speckle gratings less responsive to pressure than the standard reflection gratings. The reduction in pressure sensitivity of the speckle gratings may also be a consequence of the less structured and less uniform nature of the speckle.

### 4.4. Prototype Reflection Hologram Viewer

**Figure 13:**
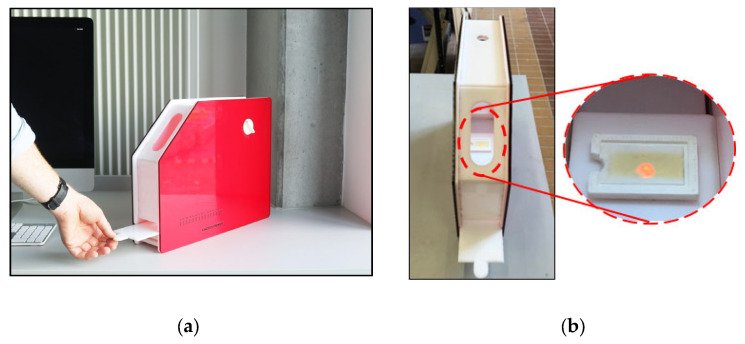
(**a**) Photograph of the exterior of the reflection hologram viewer with an adjustable sample holder; and (**b**) Photograph of the sample holder containing a reflection grating, and a close-up of the reflection grating inside the viewer.

Photographs of the fabricated reflection hologram viewer are shown below in Figure 13. A video demonstrating the operation of the reflection hologram viewer is available as part of the Supplementary Materials to this paper (Video S1: Reflection Hologram Viewer). The external walls of the viewing unit are opaque and lightproof, which minimizes the interference from external light sources. The hologram is slotted into a holder and inserted into the viewing unit. The hologram position and the rotation angle of the light source can be controlled by the user and adjusted until the reconstructed hologram colour is visible through the slit; the combination of a horizontal “slider” which holds the hologram and a rotating light source allows the user to adjust each to the correct viewing angle, in-line with the viewing slit. A graduated horizontal scale on the slider and annotated dial on the rotating light source are available so that these values can be recorded. The viewing slit size and shape allow for the user to be easily directed to the correct viewing position.

## 5. Discussion

The successful adoption of any visual colour-changing sensor or indicator technology, for any analyte or stressor, depends on many factors. One key factor is the ability of the user to see and interpret the intended colour change by eye easily and instantly. In the case of angularly selective reflection hologram-based sensors, which are fabricated in thick photopolymer layers (>100 μm), improvement in the device’s angular visibility is crucial if the sensor technology is to be viable.

Here, a holographic diffuser technique was used to improve the angular visibility of reflection holograms. The ability to record the visually bright reflection mode holographic speckle gratings in 150 μm thick films of a diacetone acrylamide-based photopolymer was demonstrated. A diffusion efficiency of 35 ± 7% was achieved for the speckle reflection gratings, comparable to a diffraction efficiency of 35 ± 2% for the standard reflection gratings; the higher uncertainty in the speckle grating efficiency is due to the diffuse nature of the reflected light. Although very similar figures are quoted for both holograms, the amount of light detected by the observer in any given direction for the reflective diffusers is smaller than that for the pure reflection grating. Via the use of appropriately sized apertures in the recording geometry, a two-fold increase in the FWHM of the reflection grating’s angular selectivity curve was achieved, in comparison to that of a standard 4500 lines/mm reflection grating (Section 4.1). The influence of the aperture dimensions on the speckle size is well understood for the transmission speckle regime. Here, the preliminary results obtained for two differently sized apertures indicate that a similar relationship between aperture size and speckle size may exist in the reflection regime. However, further studies are needed in order to further investigate this relationship in greater depth.

The FWHM of the wavelength selectivity of the device has also been approximately doubled (Section 4.2). As discussed earlier, the broadening of the wavelength selectivity of the speckle grating is further beneficial for boosting the visual brightness of the holographic devices, as a broader range of wavelengths will be reflected by the grating when illuminated with a white light source. The achievement of the high diffraction efficiencies in reflection mode may be a challenge for many holographic recording materials, due to the higher demands in terms of material spatial resolution. Speckle reflection gratings, with their broader wavelength acceptance and large feature size, may indeed offer efficiency improvements for many holographic devices.

A preliminary investigation into the ability of the speckle reflection gratings to act as holographic mechanical pressure sensors was conducted. Both the speckle and standard reflection gratings demonstrate a colour change response for a similar working range of compressional pressure (>0.88 MPa and <5.31 MPa). The speckle reflection gratings show a reduction in pressure sensitivity, which was considered a consequence of the change in polymer morphology when a speckle feature was introduced (Section 4.3). Tensile testing may indeed be a useful tool with which to further investigate the properties of speckle reflection gratings fabricated with different apertures and will be considered as part of future work.

Finally, the design and fabrication of a prototype reflection hologram viewer was presented (Section 4.4). The objective of this viewer was to minimize the challenges associated with viewing of angularly selective reflection holograms, which can act as a barrier to the adaptation of reflection hologram-based technologies. While conceptually simple, the viewer device is versatile, and described as easy to use by non-experts.

## 6. Conclusions

Here, a novel approach to improve the angular visibility of holographic reflection holograms and reflection hologram-based sensors was presented. Diffuse or speckle reflection holograms were produced by a combination of speckle recording and Denisyuk recording techniques. The fabricated holographic speckle reflection gratings exhibited broader angular and wavelength selectivity curves than standard 4500 lines/mm reflection gratings due to the formation of polymerization-induced, micron-sized speckle features within the reflection grating structure. The ability of both the standard and speckle reflection gratings to respond to compressional mechanical pressure (MPa range) via a visible colour change was demonstrated. Finally, the design and fabrication of a reflection hologram viewer which enables the straightforward viewing of angularly selective reflection holograms was also discussed. The conceptually simple viewer further opens the door for the development of reflection hologram-based sensors for a multitude of applications.

## Figures and Tables

**Table 1:** Results of the compressional pressure testing of standard and speckle reflection gratings.

Force (N)	Pressure (MPa)	Peak Wavelengths for Standard Reflection Grating (nm)			Peak Wavelengths for Speckle Reflection Grating (nm)		
		Before Pressure	After Pressure	Shift	Before Pressure	After Pressure	Shift
100	0.88	619	619	0	622	622	0
200	1.77	618	586	32	619	601	18
300	2.65	621	557	64	--	--	--
400	3.54	629	479	150	618	562	56
500	4.42	622	454	168	620	487	133
600	5.31	620	--	damage	620	--	damage

## Data Availability

Generated data available upon request.

## Supplementary Materials

The following supporting information can be downloaded at: https://www.mdpi.com/article/10.3390/s23094275/s1, Video S1: reflection hologram viewer.
